# Understanding the Bioactivity and Prognostic Implication of Commonly Used Surface Antigens in Multiple Myeloma

**DOI:** 10.3390/jcm11071809

**Published:** 2022-03-25

**Authors:** Eyal Lebel, Boaz Nachmias, Marjorie Pick, Noa Gross Even-Zohar, Moshe E. Gatt

**Affiliations:** Hadassah Medical Center, Department of Hematology, Faculty of Medicine, Hebrew University of Jerusalem, Jerusalem 91120, Israel; nachmiasb@gmail.com (B.N.); marjorie@mail.huji.ac.il (M.P.); noagros@gmail.com (N.G.E.-Z.); rmoshg@hadassah.org.il (M.E.G.)

**Keywords:** surface antigens, multiple myeloma, markers, flow cytometry

## Abstract

Multiple myeloma (MM) progression is dependent on its interaction with the bone marrow microenvironment and the immune system and is mediated by key surface antigens. Some antigens promote adhesion to the bone marrow matrix and stromal cells, while others are involved in intercellular interactions that result in differentiation of B-cells to plasma cells (PC). These interactions are also involved in malignant transformation of the normal PC to MM PC as well as disease progression. Here, we review selected surface antigens that are commonly used in the flow cytometry analysis of MM for identification of plasma cells (PC) and the discrimination between normal and malignant PC as well as prognostication. These include the markers: CD38, CD138, CD45, CD19, CD117, CD56, CD81, CD27, and CD28. Furthermore, we will discuss the novel marker CD24 and its involvement in MM. The bioactivity of each antigen is reviewed, as well as its expression on normal vs. malignant PC, prognostic implications, and therapeutic utility. Understanding the role of these specific surface antigens, as well as complex co-expressions of combinations of antigens, may allow for a more personalized prognostic monitoring and treatment of MM patients.

## 1. Introduction

Multiple myeloma (MM) is a neoplastic disease of plasma cells (PC) causing painful destructive bony lesions, anemia, hypercalcemia, kidney injury, and immune dysfunction [1]. The disease is precedented by monoclonal gammopathy of unknown significance (MGUS), a very common pre-malignant condition, characterized by a small PC clone secreting a monoclonal protein [1,2]. Though many of the abnormal properties of the PC including major chromosomal events are already present at the MGUS state, additional molecular events are needed for progression from MGUS to MM, and only a minority of MGUS patients will eventually progress to MM [1,2]. Great efforts have been undertaken to understand the pathogenesis of the evolution from a normal PC to MGUS and then MM and to understand the mechanism of MM PC survival, proliferation, and resistance to therapies [3,4,5,6,7].

Though extra-medullary disease is a known and a dismal complication of MM, the MM tumor cells in general have a high affinity to the bone marrow (BM) [8]. The expansion of tumor cells and progression of the disease within the BM is dependent on interactions between the tumor cells and the BM microenvironment, including both cellular elements and extra-cellular matrix (ECM) components [4]. This complex interaction is mediated by a network of surface proteins on both the MM cells and the BM stroma cells [9,10].

Key proteins on the MM cell surface which mediate these interactions have been an area of active research in order to reveal mechanisms for MM transformation and progression [11]. Furthermore, some of these proteins can be clinically utilized in the management of MM, initially for diagnostic purposes, but also for prognostication and lastly for therapeutic measures. Hundreds of such bioactive surface proteins have been identified and investigated, frequently including immune signaling-related proteins, followed by transporters and adhesion molecules [11].

Specific surface antigens are commonly used in flow cytometry assays for the discrimination of malignant from normal PC, diagnosis of MM and other PC dyscrasias, and evaluation of minimal residual disease (MRD) following therapy [12,13,14]. Some of these surface antigens interact with one another, and some have independent roles. Their baseline cellular functions are not always understood, and their implications on diagnosis, prognosis, and risk stratification are variable and not uniformly agreed upon.

There is a relative consensus as to which surface molecules analyzed together by flow cytometry may identify the pathogenic clone and further stratify it [15,16]. Regardless of the PC disease category, the neoplastic PC share similar immunophenotypic features that are distinct from those of normal PC. Typically, CD38 and CD138 are the backbone markers for the discrimination of PC from other cells in the BM. In addition, expression of CD45, CD19, CD56, CD117, CD28, CD27, and CD81 together with cytoplasmic immunoglobulin light-chain restriction allows for a clear discrimination between normal/reactive vs. monoclonal PC. Together, these markers are used by the EuroFlow consortium to create a standardized panel, allowing the identification and immunophenotypic characterization of the neoplastic PC [17].

This review will focus on these specific molecules, which are in routine use in many cancer centers, and will elaborate on their bioactivity, prognostic value, and therapeutic relevance. In addition, we will review the implications of the novel CD24 antigen on the pathogenesis as well as prognostication purposes in MM.

# CD38

CD38 is a surface protein which is expressed at high levels on normal and malignant PC as compared with the rest of the BM [18]. Consequently, it is utilized mostly for identification of PC, and not for discrimination between normal and malignant PC, although malignant cells can express a lower level of CD38 than normal PCs. CD38 expression is common to all hematopoietic cells but its levels are usually lower in mature myeloid cells and lymphoid lineage cells and negative on red cells and platelets. Activated hematopoietic cells upregulate CD38 expression to high levels, although not as high as PC [19]. This upregulation is due to binding to CD31 (present on the endothelium) and/or hyaluronan, which mediates CD38 adhesion to the BM ECM [8,18]. In addition, CD38 has an enzymatic activity: it can catalyze the generation of potent messengers that regulate intracellular calcium levels [20], which overall promotes proliferation. In addition, CD38-dependent adenosin production causes immune suppression. All of these bioactivities are not necessarily exclusive to PC. However, the density and high levels of CD38 on PC relative to other cells identified this protein as an optimal target for anti-myeloma therapy, and indeed, anti-CD38 antibodies have revolutionized the anti-myeloma treatment [19,20]. Lower levels of CD38 on MM cells are thought to be one of the mechanisms of extra-medullary progression of disease [21] and anti-CD38 resistance [22]; however, CD38 is not frequently used for prognostication purposes in MM.

Daratumumab, a full humanized anti-CD38 antibody, was the first anti-CD38 therapy that was investigated, followed by isatuximab [19,20]. The mechanism of both drugs involves complement-dependent cytotoxicity (CDC), antibody-dependent cell-mediated cytotoxicity (ADCC), antibody-dependent cellular phagocytosis (ADCP), induction of apoptosis, and modulation of CD38 enzyme activities [23]. Both daratumumab and isatuximab have shown unprecedented efficacy, and thus paved their way to act as the backbone of treatment of relapsed and refractory MM, in various combinations. In addition to many other advantages, the rationale for anti-CD38 combinations is the ability of some anti-myeloma therapies to increase the density of CD38 molecules on the MM cells [21]. In the last few years, daratumumab and isatuximab combinations were incorporated into frontline therapy for MM and AL amyloidosis, exhibiting valuable benefit in response rates and depths, progression-free survival (PFS), and overall survival (OS) [24,25,26,27].

# CD138

CD138 (syndecan-1) is a large glycoprotein expressed on pre-B-cells, lost during B-cell maturation and re-expressed on PC [28], where it is one of the most abundant surface molecules [29]. Consequently, similar to CD38, its role in diagnosis is mainly for the identification of PC and not the discrimination between normal and malignant PC. Carrying heparan sulphate chains, CD138 interacts directly with ECM proteins such as fibronectin, promoting cell adhesion [8]. Moreover, CD138 plays a critical role in the ability of heparan sulphate-binding cytokines and chemokines to interact with the MM cell and promote its proliferation and survival [30].

However, data suggest significant heterogeneity and plasticity in the surface phenotype of CD138 with many sub-populations within the MM PC milieu upon certain conditions [31], which include CD38+CD138^negative/low^ PC populations. Recently, CD138^high^ MM cells were characterized by a proliferative but static phenotype, as opposed to CD138^low^ MM cells which were more motile and disseminative [29]. Junctional Adhesion Molecule-C was described as a key regulator in the switch between these two states [32]. These recent findings may explain earlier reports of CD138 shedding into the plasma [33], and correlating high plasma levels of soluble CD138 with a dismal prognosis [34]. However, similar to CD38, CD138 expression in flow cytometry analysis is not widely used for prognostication. Attempts to therapeutically target CD138 have not resulted in advanced clinical development thus far [35,36,37].

# CD45

CD45 expression is ubiquitous on nucleated hematopoietic cells, and is thought to be a regulator of antigen-mediated T- and B-lymphocyte activation [38,39]. In normal BM, there appears to be a balance between the number of early PC, that express CD45, and terminally differentiated PC, that are CD45-negative [38]. In MM, the PC population is skewed toward CD45+ cells, that appear to be more proliferative compared to CD45− cells, co-express other different surface molecules, and respond differently to inhibitory and activating stimuli [38,40,41,42,43,44,45].

There is a controversy regarding the prognostic value of CD45 in MM. In newly diagnosed MM, the expression of high levels of CD45 was shown to independently predict inferior OS. CD45+ clones were thought to be a surrogate marker for a more aggressive phenotype of MM [46]. In daratumumab-treated patients, an increase in CD45 expression is associated with a very aggressive and resistant disease [47]. A small clone expressing high levels of CD45 may imply the existence of MRD that at relapse will become aggressive. In contrast, others reported worse prognosis in CD45− disease [48].

# CD19

CD19 is a B-lineage lymphocyte antigen expressed on the surface of most B-cells. Its expression appears during immunoglobulin gene rearrangement, which coincides with B-lineage commitment at the hematopoietic stem cell stage and its expression progressively increases in concentration along B-cell maturation and terminal differentiation to PC. Throughout development, the surface density of CD19 is highly regulated, with the more mature B-cells expressing higher levels of CD19 [49]. However, it is rarely present on memory cells or plasma-blasts, and is evidently present in low levels on normal PC, disappearing on malignant MM PC. Thus, it is utilized as a biomarker for B-lymphocyte development and diagnosis of B-lymphoproliferative diseases [50]. Its presence on PC helps with differentiating aberrant from normal PC. Being a B-cell marker, it has been hypothesized to be an early PC differentiation antigen and to mark the MM “stem cell”.

CD19 has a dual role. The first role is as part of the B-cell receptor (BCR) complex, allowing B-cell differentiation and the antigen-dependent maturation processes, which the cell survival is dependent on [51,52]. In the second role, it interacts with CD21 to activate the BCR, and is essential for B-cell functionality by decreasing the threshold of the BCR activation [53]. It also interacts with other ligands such as complement (C3d) receptor, CD81, and CD225 [49,54]. This causes an intra-cytoplasmatic signaling cascade through the BCR, activating PI3K, Syk, Src kinases, and AKT kinases [49,55]. CD19 also plays an active role in lymphoma pathogenesis, most intriguingly by stabilizing the concentrations of the MYC oncoprotein [54,56]. MYC is also a significant oncogene player in MM [57].

Multicentric studies have clearly shown that the phenotypic characteristics of clonal PC differ from their normal counterpart in terms of antigenic expression. The usage of antibody panels including CD19 and other aberrantly expressed markers has allowed identification of phenotypic abnormalities in MGUS and MM at diagnosis and progression of disease [15]. Interestingly, it has been published that MM patients displaying higher CD19 levels have a dismal outcome as compared with the CD19-negative patients, and expression of both CD19 and CD28 together with the absence of CD117 was hypothesized to be a marker of a MM stem cell, although there are conflicting reports as to the ability of CD19+ cells to form MM colonies in vitro [58,59].

CD19 has been utilized as a target for B-cell leukemia immunotherapies with antibodies or bi-specific antibodies, and as the main antigen used for chimeric antigen receptors (CAR)-T-cell therapy in B-cell lymphoma treatment [60,61]. The latter modality has been tested in MM patients based on the MM stem cell theory. A published case report described a deep response to CD19 CAR-T in MM patients, despite the absence of CD19 expression in 99.95% of the patient’s neoplastic PC [62]. This led to a recently reported exploration of autologous transplantation followed by CD19 CAR-T-cell therapy in MM patients [63], and moreover to the future construction of a dual anti-BCMA and anti-CD19 CAR-T strategy [64].

# CD117

CD117 (c-kit) is a tyrosine kinase receptor involved in cell differentiation and proliferation [65,66]. It is essential for the survival of CD34+ myeloid precursors, and is also strongly expressed on mast cells and some sub-populations of natural killer (NK) cells and early T-cell precursors [67]. Some cancers are also characterized by CD117 expression, including gastrointestinal stromal tumor (GIST) and lymphoproliferative and myeloproliferative neoplasms [65,67].

About a third of MM PC express CD117, as opposed to almost none of normal PC [67,68]. Interestingly, it was found that CD117 expression is frequently lost at disease relapse [65]. Similar to other malignancies, following activation by its ligand stem cell factor (SCF), CD117 promotes cell proliferation in MM [65]. Compared to CD117−, CD117+ MM patients were found to have more hyper-diploid karyotype cases, less 14 chromosome translocations, and overall, a better prognosis [65,67,68,69,70], but not in all reports [71]. C-kit inhibition was not successful as a treatment for MM [72].

# CD56

CD56, or neural cell adhesion molecule (NCAM), is a membrane glycoprotein and is a member of the immunoglobulin superfamily. CD56 is expressed on neural cells, muscle tissues, and various lymphoid cells. It is expressed on a small percent of normal PC but overexpressed in about 65–80% of malignant PC dyscrasias, especially MM [73,74,75]. Overexpression of CD56 promotes the transcription of CREB1 targets, the anti-apoptotic genes BCL2 and MCL1, resulting in a robust anti-apoptotic effect [76].

In recent years, studies assessing the relationship between MM prognosis and the expression of CD56 have been contradictory [69,77,78]. Some evidence suggests that its expression on malignant PC is a poor prognostic factor [78]. CD56 positivity in MM correlates with greater osteolytic burden, and is associated with well-differentiated neoplastic PC and a lower frequency of standard risk features, such as the presence of t(11;14) [76]. In another study, CD56 absence was associated with unfavorable prognostic parameters, such as elevated lactate dehydrogenase (LDH) and β2-microglobulin levels, advanced stage, and BM plasmacytosis of above 60%, however none of these factors effected patients’ OS [79]. Others have shown that CD56 absence may be associated with extramedullary involvement, plasma-blastic morphology, a plasma cell leukemia (PCL) state, non-hyper-diploid chromosomal abnormalities, and eventually worse PFS [65,77,80,81]. Okura et al. also reported on worse OS for CD56-negative MM [82]. In conclusion, CD56 may indeed be associated with prognosis and remains as one of the leading MM markers [83].

# CD81

CD81 is a transmembrane protein of the tetraspanin family, which is expressed on normal B-cells and plays a critical role in the regulation of B-cell receptor activation [84], and as mentioned before, trafficking and expression of CD19 [85,86,87]. In vitro studies identified an anti-tumorigenic effect of CD81 in MM cells, including reduced proliferation and invasion potential [88], as well as enhanced protein synthesis with activation of unfolded protein response (UPR) [89], causing autophagic MM cell death. CD81 has a bright expression on normal PC, and is usually dim on abnormal PC, with up to a 40–45% detection rate in MM [90,91]. Paiva et al. of the PETHEMA group reported on a cohort of 233 newly diagnosed MM patients, with CD81+ MM patients having a significantly shorter PFS (3-year rates of 26% vs. 52%, in CD81+ vs. CD81−, respectively), which translated to a significantly shorter OS [90]. This group further showed, in a larger cohort, that MM cases expressing the combination of CD38^low^, CD81+, and CD117− had the strongest correlation with an inferior outcome [92]. The significance of the CD81+ and CD117− expression pattern as a strong adverse prognostic marker was further reported by Chen et al. [68]. CD81 expression in smoldering MM (SMM) is correlated with shorter time to progression to active MM [90]. CD81 was also demonstrated as one of the most useful markers to detect different sub-clones. Interestingly, progression from MGUS to MM is characterized by reduced sub-clones’ variability with the appearance of a dominant clone. The immunophenotype profile was similar between MGUS and MM, however loss of CD27 and an increase in CD81 was noted in the dominant clone of relapsed patients [93]. A further study linked CD81 with differentiation of MM cells and identified CD19+/CD81− expressing MM cells as a more immature subset of PC [94]. Thus, there is a discrepancy between the ex vivo findings of lower tumorigenicity and the expression levels correlating with poor prognosis.

# CD27

CD27 is a membrane glycoprotein of the tumor necrosis factor (TNF) superfamily, which is expressed on the surface of most peripheral T-cells and certain sub-populations of B-cells [95], specifically memory B-cells and PC [96]. CD27 expression on T-cells acts as a co-stimulatory receptor, while binding of CD70 to CD27 on B-cells promotes differentiation to PC [97]. Several studies have shown that loss of CD27 expression characterizes progression to MM and less favorable prognosis [93,98,99]. Chu et al. investigated the significance of CD27 expression in newly diagnosed MM patients and found that CD27-negative disease had higher adverse risk characteristics, including higher PC burden and advanced stage. Furthermore, PFS was significantly shorter in the CD27-negative group [100]. Counterintuitively, the tumor cells in PCL displayed a high expression of CD27, which was shown to be protective of dexamethasone-induced apoptosis [101]. The difference between MM and PCL regarding CD27 expression is still not well understood.

# CD28

CD28 is expressed on T-cells and acts as a co-stimulatory receptor with the T-cell receptor, resulting in enhanced proliferation and cytokine secretion [102,103]. CD28 expression is highly specific for MM PC, as it is not expressed on normal PC. Moreover, CD28 expression is correlated with disease progression, reaching up to 93% and 100% expression on relapsed extra-medullary MM and PCL, respectively [104]. T-cell activation is mediated by the binding of CD80 and CD86, expressed on antigen-presenting cells, to CD28 on MM PC. Data suggests that CD80/86 binding to CD28 on MM PCs generates a crosstalk between the stromal and MM PC. This promotes secretion of IL-6 by the stromal cells, which in turn supports survival of MM cells and ameliorates the anti-proliferative effects of dexamethasone [105]. In addition, activation of CD28 also induces activation of PI3 kinase signaling in MM PC, with downstream nuclear factor kappa B (NFkB) activation causing a pro-survival effect, which further strengthens the role of CD28 in the stroma–myeloma cell interaction [106].

# CD24

CD24 is a highly glycosylated protein expressed on the surface of most B-lymphocytes, neutrophils, and its precursor myelocytes and differentiating neuroblasts. CD24 is expressed on pre-B-lymphocytes, remains expressed on mature resting B-cells, and becomes downregulated during the maturation process to PC [107,108]. A lack of CD24 results in a decrease in maturation of B-cells in mice [109]. CD24 overexpression has been found in many solid cancers and has been correlated with worse prognosis and presence of metastasis [110,111,112]. In MM PC, CD24 mRNA has been shown to be downregulated, correlated to worse OS [12]. We found that by upregulating CD24 expression in MM cell lines, the cells were less tumorigenic in their phenotype, as assessed by their impaired capability to migrate and to create colonies in culture [113]. The decreased tumorigenicity correlated with a “more normal” PC immunophenotype in patients with MM and correlated with CD45 expression [113]. Furthermore, following immunophenotype analysis in 124 MM patients treated with bortezomib, cyclophosphamide, and dexamethasone, we found that elevated CD24 expression on PC at diagnosis correlated significantly with longer PFS and OS (Gross Even-Zohar N, et al., submitted). Indeed, among CD19, CD45, CD117, CD56, and CD24, the latter was the only marker that retained its prognostic impact on PFS and OS. In light of these results, we believe that the addition of CD24 to the immunophenotype panel of MM at diagnosis should be considered. Its expression during the course of the disease is being studied.

## 2. Summary

In this review, we have highlighted the significance of selected surface antigens for the diagnosis and prognostication of MM, as well as in vitro data supporting their implication on disease course and outcomes, and their therapeutic utility. These aspects are summarized in Table 1.

Among the variety of surface antigens, we chose to focus on the markers which are commonly used for the detection of aberrant PC in flow cytometry [12,13]. Most were also implicated at prognostication, and therefore are commonly reported in routine clinical practice. However, the great interest in these molecules stems from a rationale which extends way beyond practical measures, as these molecules in some way can “tell the story” of MM progression and dissemination.

Normal PC express CD38^bright^, CD138, CD27, CD19, and CD81, but usually do not express CD56 and CD117 [68]. MM cells also express CD38^bright^ and CD138, however they lose the fine balance of CD45+ and CD45− cells which characterize the normal PC milieu [38]. Moreover, they tend to lose CD27 and CD81, and in some cases acquire CD28, CD56, and CD117 [68]. Though only a very small proportion of MM cells express CD19, this sub-population is thought to represent the MM stem cells, which contribute to clonal selection of immature, aggressive, and resistant tumor following anti-myeloma therapy.

The above surface antigens are also very important in the discrimination between MGUS, SMM, and active MM. In general, the abnormal PC of MGUS and SMM usually share the same abnormal immunophenotype of MM PC. However, it is the percentage of normal vs. abnormal PC which is helpful in the discrimination between these different disorders. In virtually all MGUS cases, a normal PC population can be found, which rarely constitutes less than 3–5% of the total PC population. In contrast, in only 1.5% of active MM cases, a normal PC population of more than 3–5% of total PC exists. Accordingly, in SMM, the percentage of normal PC can be informative with regards to progression risk—whether it is closer to the MGUS state or to the active MM state [13,15]. Similarly, the minority of MM cases with >5% of normal PC were found to have favorable outcomes [15].

As reviewed here, the expression of certain surface antigens signifies a specific tumor biology, which correlates with disease outcomes. The expression of both CD38 and CD138 is common to all PC, including normal and MM PC, where they serve as adhesion molecules, have enzymatic activity, and promote proliferation by binding cytokines. However, CD38^negative/low^ clones and CD138^negative/low^ clones do exist in MM and are associated with dissemination of the disease and extra-medullary disease [21,29]. Anti-CD38 antibodies have revolutionized anti-myeloma therapy, utilizing the density of these antigens on MM cells’ surface.

CD19 is a pan-B-cell antigen with low expression on MM PC. It functions as part of the BCR complex activation and B-cell stimulation and differentiation. CD19 serves as a major surface marker in flow cytometry to identify normal from aberrant PC, and elevated levels of CD19 on active MM cells have been correlated to worse prognosis [114]. Being an early B-cell marker, CD19 has been targeted for anti-CD19 CAR-T-cell therapy for eradication of myeloma-propagating cells [64]. CD81 is involved in the regulation of BCR and CD19, and similar to CD19, its expression in MM is associated with a dismal prognosis [92].

Both CD27 and CD24 play a role in B-cell maturation and differentiation to PC and are usually expressed on normal PC. In MM, the expression of both CD27 (which occurs rarely) and CD24 (variable) is associated with favorable prognosis [12,98].

CD56 was initially described as an adhesion molecule, however recently its role in the induction of anti-apoptotic proteins was described [76]. Acquisition of this molecule in MM is associated with worse prognosis in most but not all reports [76]. Acquisition of CD28, which plays an important role in the interaction between MM cells, stromal cells, and immune cells, is also associated with progressive disease [104].

Some single antigen expressions are interestingly associated with specific major cytogenetic alterations (i.e., CD117) [114]. However, the complexity of the tumor biology goes beyond the expression of a single antigen. In some cases, it is the combination or co-expression of a few antigens that better accounts for phenotypic differences in different MM cases and consequently better prediction of prognosis [12,114,115].

Finally, one should take into account the intra-tumoral sub-populations, with possible different immunophenotypes and different clonogenic and dissemination potential. For example, circulating MM tumor cells are characterized by lower expression of adhesion molecules (CD138, CD56, CD117, and others) and activation molecules (CD38, CD28, and CD81) [116,117].

In conclusion, the MM surface antigens that were reviewed here are utilized routinely for MM diagnosis and monitoring. Understanding their bioactivity, prognostic value, and therapeutic potential can assist in the refinement of personalized MM care.

## Figures and Tables

**Table 1:** Selected key surface antigens on MM cells *.

	Cellular Activity	Expression on Normal PC	Expression on MM Cells	Prognostic Value of Expression	Therapeutic Applications
**CD38**	Adhesion, enzymatic activity (calcium regulation)	+++	+++	Low-level clones are associated with extra-medullary disease	Anti-CD38 antibodies are among the most important therapies
**CD138**	Adhesion, binding cytokines, and promoting proliferation	+++	+++	High levels signify proliferative clones, low levels signify disseminative clones	Anti-CD138 antibodies not successful
**CD45**	Promotes proliferation and activation	Balanced CD45+/CD45- PC milieu	++	Controversial	Not developed
**CD117**	Promotes proliferation	_	30%	Favorable on most reports	Imatinib not successful
**CD19**	differentiation and activation of B-cells	+ (low proportion)	Absent (but present on MM stem cells)	Adverse	CD19 CAR-T/combined BCMA/CD19 CAR-T is promising
**CD56**	Adhesion, induction of anti-apoptotic proteins	_	65–80%	Controversial	Not developed
**CD28**	Interaction with stromal support cells and T-cells	_	++	Adverse, commonly expressed in aggressive progressions	Not developed
**CD27**	Differentiation from B-cell to PC	++	Usually absent	Favorable (but usually absent)	Not applicable
**CD81**	Regulation of BCR and CD19	++	40–45%	Adverse	Not developed
**CD24**	B-cell maturation	+	Variable	Favorable even in low levels	Not applicable

Abbreviations: PC, plasma cells; MM, multiple myeloma; BCR, B-cell receptor; BCMA, B-cell maturation antigen; CAR-T, chimeric antigen receptor T-cell. * Note: All the data presented in the table are discussed in the text. The references are cited in the relevant sections of the text. +++ means high expression, ++ lesser expression. + lessser expression.
